# A Case Study of ADHD Management in a National-Level Athlete: Achieving Balance Between Academics and Sports Performance

**DOI:** 10.1192/bjo.2025.10739

**Published:** 2025-06-20

**Authors:** Praveen Kumar, Emilia Jamil, Sheena Shah, Kevin Edassery

**Affiliations:** 1 City Hospital, Aberdeen, United Kingdom; 2 Kildare West Wicklow, Kildare Town, Ireland; 3 Britton House (CMHT) Gillingham, Kent, United Kingdom

## Abstract

**Aims:** This case study explores the assessment and management of ADHD in a 14-year-old patient seen in CAMHS, presenting with significant academic difficulties but excelling as a national-level athlete in netball. The challenges of balancing stimulant treatment for ADHD with optimal sports performance highlight the need for personalised care strategies.

**Methods:** A 14-year-old patient was referred to CAMHS for concerns regarding persistent inattention, poor focus, and declining academic performance. A neurodevelopmental assessment, including the NICHQ Vanderbilt and ACE scales, confirmed a diagnosis of ADHD (combined type). Symptoms such as difficulty concentrating on tasks requiring sustained mental effort, poor organisational skills, and a tendency to rush through assignments without completing them were particularly prominent. These difficulties impacted her performance in core subjects, including mathematics, science, and English, where her grades had significantly declined.

Despite academic challenges, the patient demonstrated exceptional athletic abilities, excelling as a netball player at the national level. Her coach praised her spontaneous, quick decision-making and high energy, attributes she associated with her ADHD.

After discussion, the patient was initiated on stimulant medication (methylphenidate). Following treatment, her focus, organisation, and overall academic performance improved, with notable achievements in her exams. However, the patient and her coach reported a decline in her sports performance, attributed to the loss of the “ADHD edge”, a concept supported in literature that highlights how ADHD traits, such as hyper-focus and spontaneity, can be advantageous in certain sports contexts. The patient felt her creativity and spontaneity, critical to her athletic success, were diminished.

**Results:** In collaboration with the patient, her family, and her coach, a flexible management plan was devised. The patient agreed to withhold methylphenidate on sports days while maintaining its use during school days. This approach allowed her to excel academically while preserving her peak performance in sports, achieving the best of both worlds.

**Conclusion:** This case highlights the nuanced challenges of managing ADHD in high-performing athletes. The combination of stimulant medication with a tailored regimen offers a balanced solution, enabling optimal academic and athletic outcomes. Further exploration into the interplay between ADHD and sports performance may guide future management strategies for similar cases.

